# *The power of zinc*: excess and deficiency of Zn decrease cannabinoids in cannabis without Zn toxicity concerns to consumers

**DOI:** 10.1186/s42238-026-00404-0

**Published:** 2026-03-05

**Authors:** Sivan Shiponi, Nirit Bernstein

**Affiliations:** Institute of Soil Water and Environmental Sciences, Volcani Institute, 68 HaMaccabim Road, P.O.B 15159, Rishon LeZion, 7505101 Israel

## Abstract

**Background:**

Some of the mineral nutrients essential for plants are heavy metals, which their consumption may involve health concerns to the consumers. Hence, for safe consumption, optimized fertilization protocol for cannabis plants needs to focus also on minimizing the accumulation of potentially toxic minerals in the inflorescences. Zinc is an essential microelement for plants that has a toxic effect on the human body when consume in high concentration. The present study aimed to understand the drug-type (medical) cannabis plant response to Zn supply, for identifying the optimal Zn supply that balance high quality production with safe product.

**Methods:**

cannabis plants were grown under five Zn levels (0.05, 0.1, 0.35, 1.0, 4.0 mg L^− 1^) in controlled environment; and morpho-physiology analyses, cannabinoid profile, and ionome-profiling in the plant-organs were conducted.

**Results:**

Increased level of Zn supply reduced the relative accumulation of Zn in the inflorescences, and excess Zn was stored in the plant root, and therefore does not impose additional health risk to consumers. Cannabinoid concentrations were highest under 0.35 mgL^− 1^ Zn supply, and decreased with further increase in Zn supply. The acidic cannabinoids THCA, CBDA, CBCA, CBDVA, THCVA increased with the increase in Zn supply up to 0.1–0.35 mgL^− 1^ and declined with further increase in Zn. Zn deficiency (under 0.05 mgL^− 1^ Zn supply) reduced physiological performance, plant growth and inflorescence yield, and stimulated uptake of Zn, P, S, Ca, Fe, and Mn. Symptoms of excess Zn were death of leaf tips; however plant performance was overall not affected by Zn excess.

**Conclusions:**

Excess Zn is retained in the root and excluded from the inflorescence, thereby not imposing health risk to the consumers. The recommended Zn concentration in the fertigation solution that was found to attain highest specialized-metabolite concentrations, and optimal yield and plant performance is 0.35 mgL^− 1^.

## Introduction

Cannabis is a rising commercial high-value crop. However, prohibition and legal restrictions on research resulted in limited understanding of the plant science (Bernstein et al. 2019b; Monthony et al. 2021). Recently, the cannabis medical and recreational industries are growing exponentially and with it the development of regulations for safety and quality of the plant produce (Bahji and Stephenson 2019). Science-based knowledge of the crop is required for developing agro-techniques for production of safe and high-quality medical plant product. The pharmacological effects of cannabis are attributed to its specialized secondary-metabolites, including terpenoids, flavonoids and the cannabis-specific cannabinoids, which are produced and stored to the highest concentrations in trichomes on the female inflorescences. In cannabis, similar to other plants, secondary metabolite production is affected by genetic and environmental conditions. Cannabinoid concentrations were found to be affected by a range of exogenous factors including mineral nutrition (Bernstein et al. 2019b; Bevan et al. 2021; Saloner and Bernstein 2021b; Shiponi and Bernstein 2021b), stress conditions (Song et al., 2023), salinity (Yep et al., 2020), drought (Caplan et al. 2019), light spectrum (Desaulniers Brousseau et al. 2021), plant architecture (Danziger and Bernstein 2021), growing media (Caplan et al. 2017), and temperature (Chandra et al. 2011).

Nutrient supply is essential for plant survival and development. The essential nutrients are required for the plant in varying amounts, and are commonly divided into macro- and microelements based on the concentrations found in the plant tissues. While information was recently published on response of the drug-type cannabis plant to some macronutrients, responses to micronutrients is yet to be studied. Supply of the three major macronutrients N, P and K was found to affect the cannabis plant morpho-physiology and mineral distribution in the plant body at both the vegetative (Caplan et al. 2017; Saloner et al. 2019; Shiponi and Bernstein 2021a; Kpai et al. 2024) and the reproductive stages of development (Bernstein et al. 2019b; Bevan et al. 2021; Saloner and Bernstein 2021b; Shiponi and Bernstein 2021b). Generally, cannabinoids concentration was found to decrease with the increase of N, P and K concentration in the nutrient solution. While some work has been done on industrial hemp’s microelements requirements (Cockson et al., 2019; Wylie et al. 2021; Berni et al. 2024), little information is available on drug-type cannabis.

Zinc is an essential microelement that functions as a cofactor for hundreds of proteins. It involves in defense against oxidative stress, plays an important role in membrane permeability and stability, regulates conformational stabilization of several proteins and take part in hormone regulation (Brown et al. 1993; Tsonev and Lindon 2012; Gupta et al. 2016). Therefore, Zn participates in numerous key physiological processes, and attaining information on its effect on physiological function and secondary metabolism in cannabis is of importance for optimizing yield quality and quantity. Furthermore, Zn is an essential element for the human body as well, however exposure to high quantity of Zn imposes health risks for humans and there is a rising concern about the safety of medicinal cannabis due to potential accumulation of Zn and other heavy metals in the plant product (McPartland and McKernan 2017; Sarma et al. 2020). This concern is heightened by the realization that fiber-type (Hemp)-cannabis is a good bioaccumulator of heavy metals and can be used for phytoremediation of polluted soils (Zielonka et al. 2020). Thus, understanding medical cannabis bioaccumulation behavior of Zn is important for regulation and precision of the cultivation technics in an attempt to achieve a safe product for the consumers.

Microelements, including Zn, haven’t been tested for their influence on the phytocannabinoids contents, and the physiology, morphology and biomass production in medicinal cannabis. Furthermore, bioaccumulation of microelements and their effect on plant ionome haven’t been studied in drug type cannabis. To fill the knowledge-gap the following hypothesis were tested in the present study: (i) Zinc supply affects cannabinoid concentrations in the inflorescence. (ii) Zn-imposed changes to the cannabinoid profile are accompanied by changes to the plant ionome and morpho-physiology. To test the hypotheses medical-‘drug-type’ cannabis plants were exposed to 5 Zn concentrations (0.05, 0.1, 0.35, 1, 4 mgL^− 1^) and chemical, visual, physiological and morphological responses were studied. The achieved information is fundamental for development of optimal precision agro-fertigation practices for attaining a safe, high quality medicinal product, along with the contribution for understanding of mineral nutrition regulation of cannabinoids production.

## Materials and methods

### Plant material and growing conditions

Cuttings from a single mother plant of the medicinal cannabis cultivar ‘Annapurna’ (Canndoc LTD, Israel) that contain ~ 9% CBD, ~ 9%THC were rooted in coconut fiber plugs (Jiffy International AS, Kristiansand, Norway), with the aid of the rooting hormone Indol Butiric Acid (Hormoril 3, Gadot-Agro, Ashdod, Israel), under 25 °C. 90% humidity, and a 16:8 light/dark photoperiod. Following 14 days of rooting, the rooted cuttings were transplanted to 3 L pots in a perlite growing medium (2-1-2, Agarkel, Bonim, Israel) in a controlled environment growing room at 25 °C and 60% air relative humidity. The transplanted plants, were divided randomly to 5 Zn treatment groups: 0.05, 0.1, 0.35, 1, 4 mgL^− 1^ (0.765, 1.529, 5.353, 15.295, 61.180, mM)), 6 replicated plants per treatments and were grown under a long photoperiod (18:6 h light/dark) at 400 µmol m^− 2^ s^− 1^ using Metal halide bulbs (Solis Tek Inc, Carson, California; 25.9 mol m^− 2^ d^− 1^). Fourteen days after transplanting and initiation of the Zn treatments, a short photoperiod (12/12 hr light/dark) was applied to induce flowering using High Pressure Sodium bulbs (980 µmol m^−2^s^− 1^, Greenlab by Hydrogarden, Petah Tikva, Israel) and cultivated under these conditions for 57 days. The plants were irrigated with 1 L h^− 1^ discharge-regulated drippers (Netafim, Tel-Aviv, Israel), 1 dripper per pot. The volume of irrigation in each daily irrigation event was regulated to produce 30% drainage. The plants were exposed to the Zn treatments until harvest, 57 days after the transition to the short photoperiod. Zn was applied as Zn-EDTA (Haifa Micro Zn14, Haifa Group, Haifa, Israel). The nutrient solution also contained (in mM): 10.42 N-NO_3_^−^, 2.07 N-NH_4_^+^, 1.94 P-PO_4_-^2^, 2.56 K^+^, 2.99 Ca^+ 2^, 1.44 Mg^+ 2^, 1.47 S-SO_4_^−2^, 0.06 Cl^−^, 0.021 Fe^+ 2^, 0.011 Mn^+ 2^, 0.009 B^+ 3^, 0.0008 Cu^+ 2^, 0.0003 Mo^+ 2^. Micronutrients were supplied chelated with EDTA (Cu, Mn, Mo, Zn), EDDHSA (Fe) (Barkoret, ICL, Haifa, Israel), and B was supplied as B-7000 (ICL, Tel-Aviv, Israel). pH of the fertigation solution was adjusted to 6.0 using a pH meter (Cyberscan pH 1500, Eutech Instruments Europe B.V., Nijkerk, Netherlands). Prior to planting the perlite-filled pots were irrigated twice daily, for two days, with a full fertigation solution, in a volume adjusted to generate 35% leachate; and a preliminary measurement showed that Zn concentration in the leachates following this saturation period were 99 − 98% of the concentrations in the treatment solutions. Routine analyses of the irrigation solutions confirmed that Zn concentrations were according to the planed treatment concentrations.

### Plant development

Plant height was measured once a week throughout the experiment from the base of the plant to the top of the main stem. On the termination of the experiment, 57 days following the transition to short-day (71 days after the initiation of the Zn treatments, and the transplanting the rooted-cuttings to the experimental pots) the number of nodes as recorded, and stem-diameter was measured with a digital caliper 1 cm from the base of the plant. At harvest, each plant was manually separated to leaves, stems, root, inflorescences and inflorescence-leaves, each sample was weighted for fresh weight, rinsed twice in deionized-water and dried in 65˚C for 72 h prior for weighing for dry weight determination.

### Inorganic mineral analysis

The dried inflorescences, leaves, stem and root samples were ground to a powder, and two different procedures were used for extraction of the elements from the plant tissue. For the analysis of Mg, Mn, Ca, Zn, and Fe, the ground tissue was heat-digested with the acids HNO_3_ (65%) and HClO_4_ (70%). For the analysis of N, P, and K, the ground tissue was acid heat-digested with H_2_SO_4_ (98%) and H_2_O_2_ (70%) (Bernstein et al. 2005). Mineral analysis was performed by dual-view High-Resolution ICP-OES spectrometer PlasmaQuant PQ9000 for P, K, S, Ca, Mg, Fe, Mn, Zn, Cu. Nitrogen was analyzed using autoanalyzer (Lachat Instruments, WI, USA).

A bioaccumulation coefficient factor (BCF) (L Kg-^1^), and a translocation factor (TF) of minerals were calculated by Eqs. 1 and 2, and represent the uptake (BCF) of individual nutrients into the plant, and their root to shoot translocation (TF).

1 $$BCF=\frac{Concentration\:of\:the\:mineral\:in\:the\:plant}{Concentration\:of\:the\:mineral\:in\:the\:solution}$$

2 $$TF=\frac{Concentration\:of\:the\:mineral\:in\:the\:shoot}{Concentration\:of\:the\:mineral\:in\:root}$$

### Physiological parameters

To characterize the physiological state of the plants, physiological measurements were conducted on day 37 and 57 following the initiation of the Zn treatments (5 and 2 weeks before harvest). At each measurement, two fully (youngest) expended fan leaves were sampled from each plant, and one leaf was immediately weighted for relative water content (RWC) analysis, washed twice in distilled water and gently blotted dry, and placed in a 50 ml test-tube filled with distilled water for 24 h at room temperature. Following washing in distilled water, the central leaflet of the second leaf was placed in 50 ml test-tube filled with 30 ml distilled water and was shaken for 24 h for membrane leakage measurement (Shiponi and Bernstein 2021a). For photosynthetic pigment analysis, five discs 0.6 cm in diameter were sampled from the leaf and placed in 0.8 ml 80% (v/v) ethanol in an Eppendorf tube and kept in −20 °C until further analysis. Further analysis was conducted as described by Saloner et al. (2019). Gas exchange parameters (Photosynthesis, stomatal conductance, transpiration rate, intercellular CO_2_ concentration) were measured by a Licor 6400 XT system (LI-COR, Lincoln, NE, USA), (CO_2_ concentration: 400 mg L^− 1^, PPFD: 500 µmol (m^2^s)^−1^). Measurements were conducted twice during the experiment, on day 36 and 56 after the transition to the short-photoperiod, 1 h after irrigation and lights turn-on. The measurements were conducted on the youngest fully mature fan leaf of each replicated plant.

### Cannabinoid analysis

The plant material was sampled for cannabinoid analysis at the termination of the experiment. The apical inflorescence of the main stem (primary inflorescence) and the apical inflorescence from the lowest branch on the main stem (secondary inflorescence) were cut from the plants, hand trimmed and dried in an environment-controlled chamber in the dark at 19.5 °C and 45% air humidity for 2 weeks. For the cannabinoid analysis, 50 mg of manually ground, dried inflorescence was extracted in 10 ml ethanol ABS AR (Gadot-Group, Netanya, Israel), in a 20 mL scintillation vial. The vials were shaken in a reciprocal shaker for 1 h at room temperature and filtered through a polyvinylidene difluoride (PVDF) membrane filter of 0.22 μm pore size (Bar-Naor ltd, Ramat Gan, Israel). The analysis was conducted as described by (Shiponi and Bernstein 2021b) using a high-performance liquid chromatography (HPLC) system (Jasco 2000 Plus series, Easton, MD, USA) in a spectrum mode. Luna Omega 3 μm Polar C18 column (Phenomenex, Torrance, CA USA) was used for Chromatographic separations in the isocratic mode with 75:25 (v/v) acetonitrile: water and 0.1% formic acid, at a flow rateof 1.0 mL min^− 1^. The detection was carried out in a spectrum mode, at the wavelength range 200–650 nm. Quantification was based on analytical standards: tetrahydrocannabivarinic acid (THCVA), cannabinol (CBN), cannabichromene (CBC), cannabichromenic acid (CBCA), cannabidivarin (CBDV) cannabidiol (CBD), cannabidiolic acid (CBDA), cannabichromevarin (CBCV), cannabidivarinic acid (CBDVA), cannabigerol (CBG), cannabigerolic acid (CBGA), cannabinolic acid (CBNA), cannabicyclol (CBL) (Sigma-Aldrich, Germany) and tetrahydrocannabinolic acid (THCA), Δ^9^-tetrahydrocannabinol (THC), tetrahydrocannabivarin (THCV) (Restek, Pennsylvania, USA). CBN, CBDV, CBCV, CBNA and CBL were below the detection limits.

### Statistical analysis

The data was statistically analyzed by one-way or two-way ANOVA, followed by a post-hoc Tukey HSD test (*α* = 0.05). Comparison of relevant means was conducted using Fisher’s least significant difference (LSD) test at 5% level of significance. The analysis was performed with the Jump software (Jump package, version 14 (SAS 2018, Cary, NC, USA).

## Results

### Morphology, biomass and visual symptoms

**Fig. 1:**
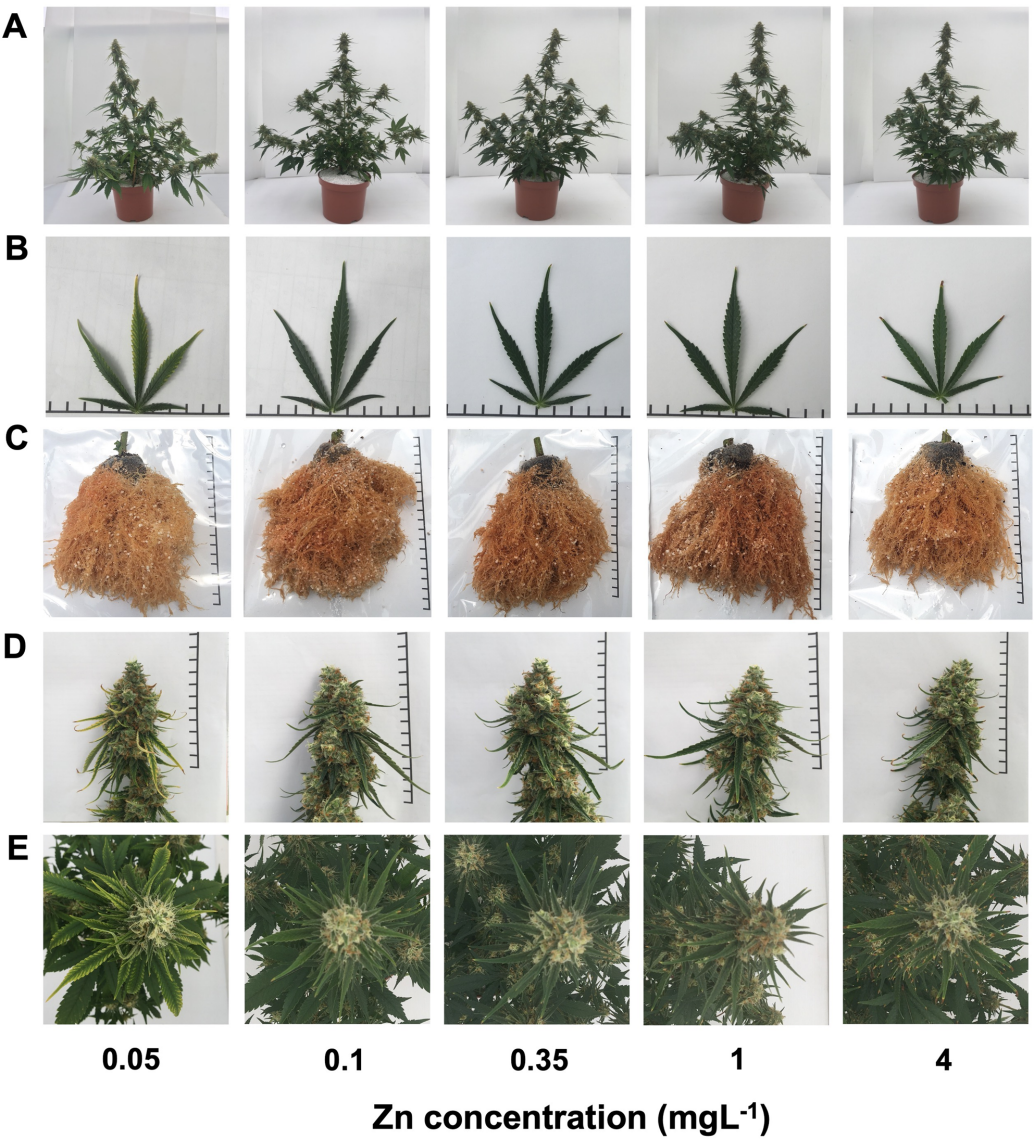
Response of medical cannabis plants to Zn supply. Visual appearance of whole plant (**A**), leaves (the youngest fully developed leaf on the main stem) (**B**), root (**C**) and inflorescences (apical inflorescence of the main stem) (**D**, **E**) under 0.05, 0.1, 0.35, 1, and 4 mgL^− 1^ (ppm) Zn. In C, the brown portion at the base of the roots is a remnant of the coconut-fiber plug that the cuttings were rooted in, and the white particles on the root are remaining perlite particles of the soil-less cultivation culture. The scale bar is a measurement of 1 cm spaces. The images were taken at plant maturity

**Fig. 2:**
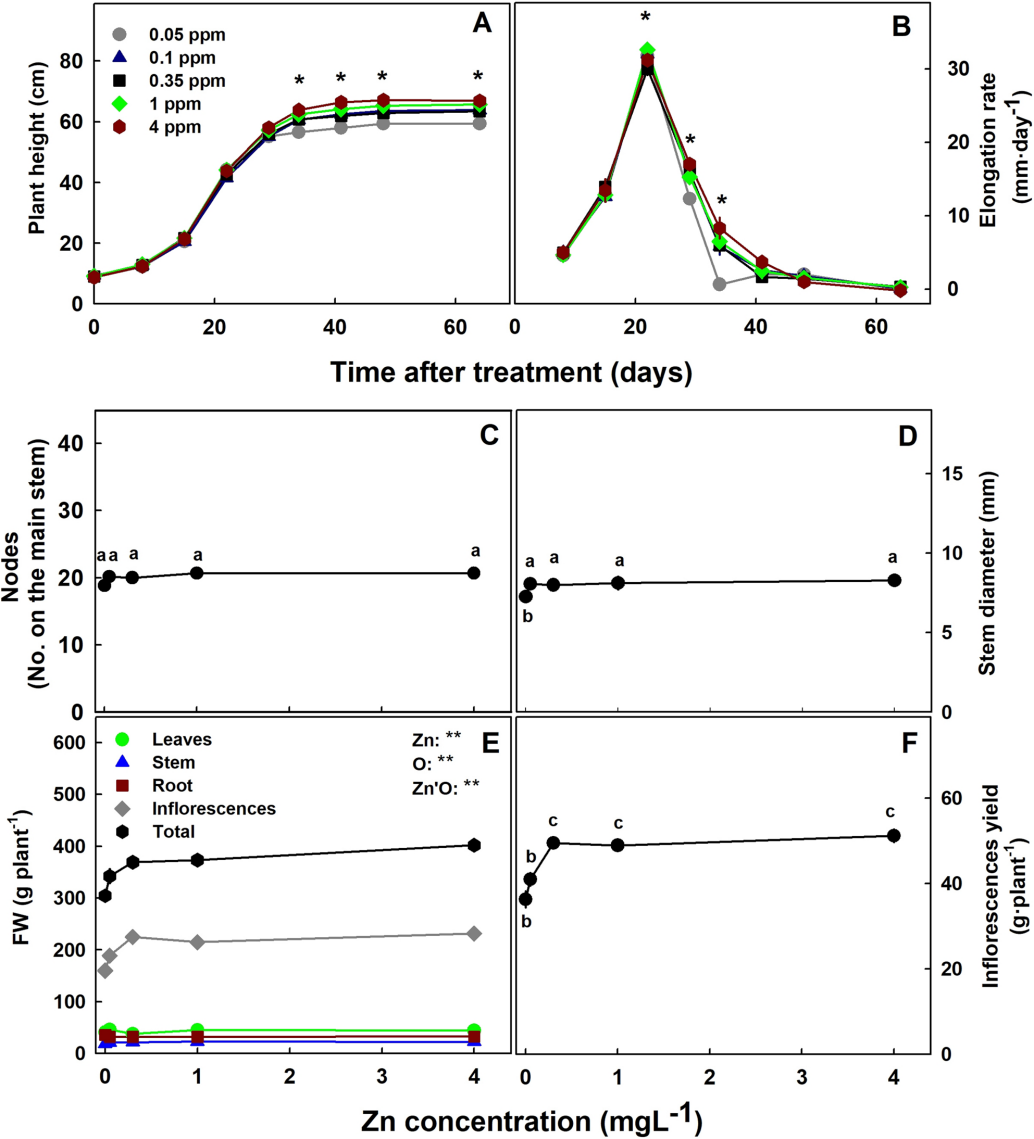
Effect of Zn concentration on the development of medical cannabis plants at the reproductive growth phase. Plant height (**A**), Elongation rate (**B**) Number of nodes on the main stem (**C**), Stem diameter (**D**) dry weight (**E**) and inflorescence yield (DW) (**F**). Presented data are averages ± SE (n=6). Data for **C**-**E** were taken at the termination of the experiment. In **A**-**B**, asterisk above the averages represent significant differences between treatments for a given day (a = 0.05). In **C**, **D**, **F** different letters represent significant differences between treatments by Tukey HSD test at a = 0.05. In **E**, Results of two-way ANOVA indicated as ** *P* < 0.05, F-test; NS, not significant *P* > 0.05, F-test. In the ANOVA results Zn is Zinc, O is organ, and Zn'O represents the interaction between Zn and O

The level of Zn supplied to the plants had but a small effect on plant development and biomass accumulation. Zn deficiency, in the form of interval chlorosis (“mottled leaf”) was noticeable in young leaves of plant grown under the lowest Zn level of 0.05 mgL^− 1^ Zn (Fig. 1B, C,E). Under this deficiency level the plants were shorter (Figs. 1A and 2A), had lower total biomass (Fig. 2E), their elongation rate was repressed by day 29 (Fig. 2B), and stem diameter was smaller (Fig. 2C, D). The lowest biomass accumulation under Zn deficiency was due to a reduction in inflorescences yield in the 0.05–0.1 mgL^− 1^ range. The number of nodes on the main stem was not affected (Fig. 2C). Further increase in Zn supply did not affect the plant morphology and biomass. Leaves, stem and root biomass were not affected significantly by the Zn treatments. Zn toxicity was visually apparent in plants that received the highest Zn concentration of 4mgL^− 1^ Zn as tissue death at the tip of the leaves (Fig. 1B, C,E).

### Cannabinoids

**Fig. 3:**
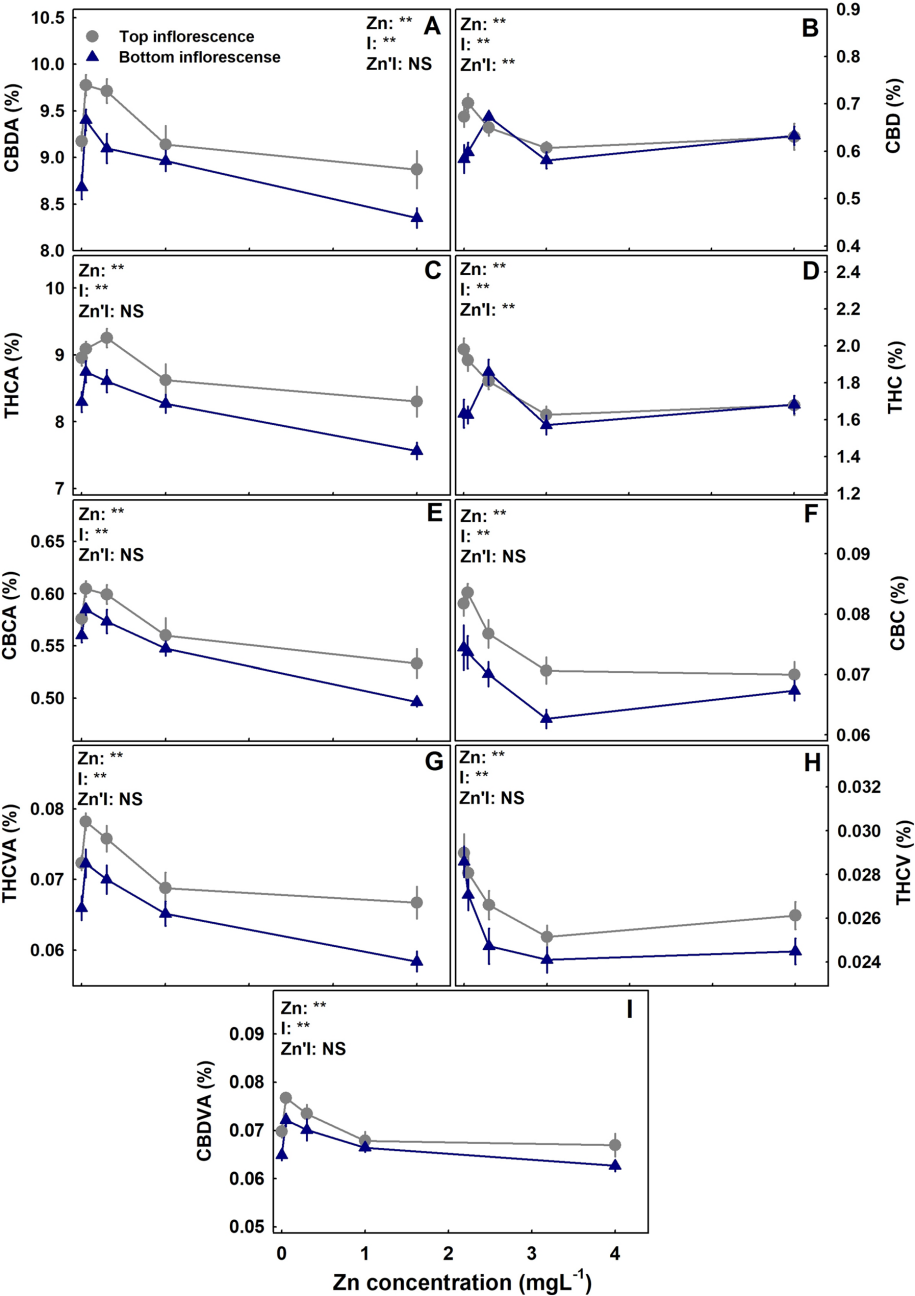
Effect of Zn supply on cannabinoid concentrations in top and bottom **inflorescences** in medical cannabis plants. CBDA (**A**), CBD (**B**), THCA(**C**), THC (**D**), CBCA (**E**), CBC (**F**), THCVA (**G**), THCV (**H**), CBDVA (**I**). Presented data are averages ± SE (*n* = 6). The inflorescences analyzed were the apical inflorescence of the main stem (top inflorescence) and of the bottom branch on the main stem (Bottom inflorescence). Results of two-way ANOVA indicated as NS (not significant) *P >* 0.05, ** *P* *<* 0.05, *F*-test. In the ANOVA results Zn`I represent the interaction between Zn and inflorescence location

**Fig. 4:**
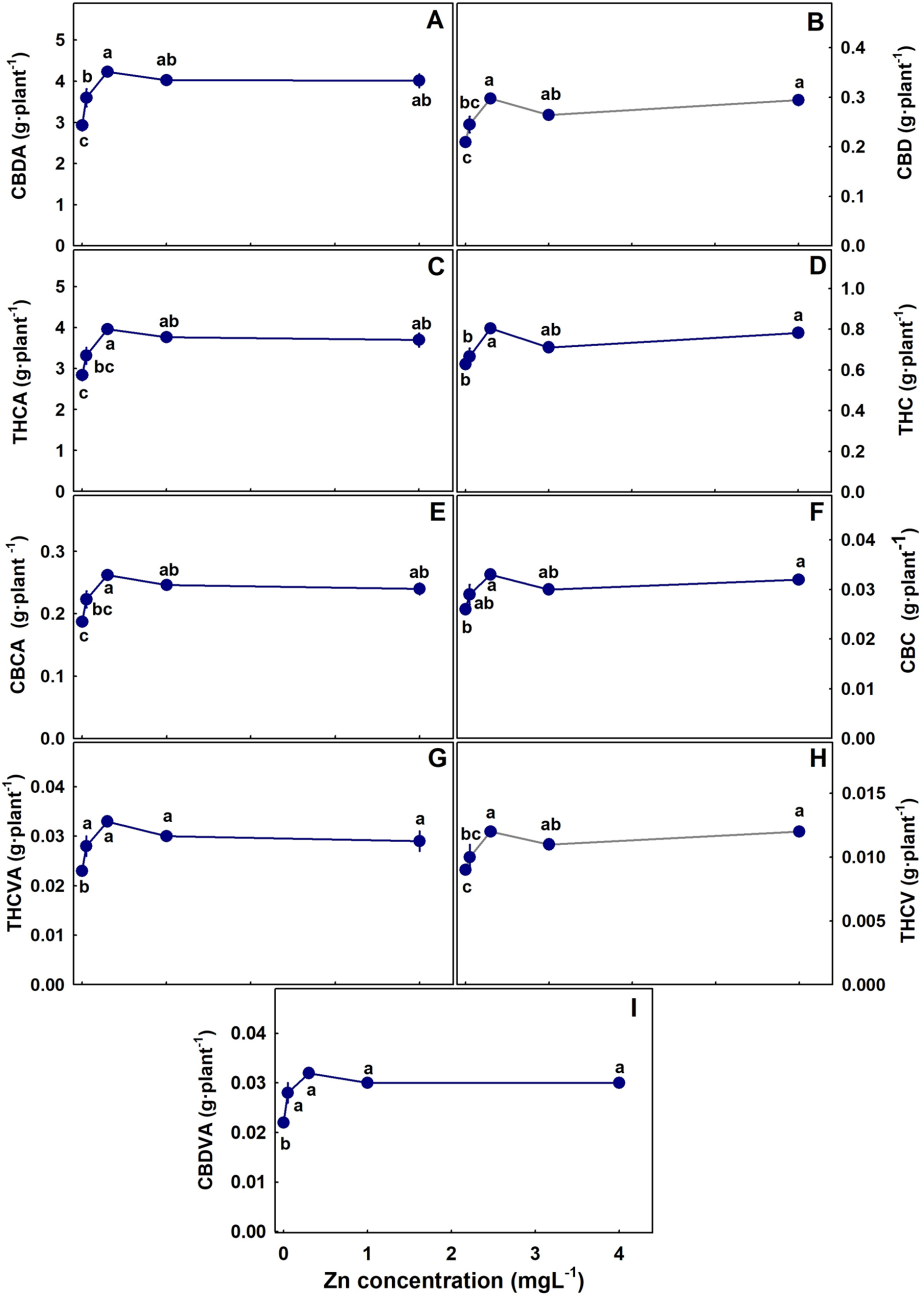
Effect of Zn application on **cannabinoid yield per plant**. CBDA (**A**), CBD (**B**), THCA(**C**), THC (**D**), CBCA (**E**), CBC (**F**), THCVA (**G**), THCV (**H**), CBDVA (**I**). Presented data are averages ± SE (*n* = 6). Different letters above the means represent significant differences by Tukey HSD test (α = 0.05)

The level of Zn supplied to the plants had a considerable effect on the concentration of cannabinoids in the inflorescence (Figs. 3). In both the primary and the secondary inflorescences, the concentration of all the acidic cannabinoids tested (CBDA, THCA, CBDVA, THCVA and CBCA) demonstrated a maximum response curve; they increased with the increase in Zn supply up to 0.1–0.35 mgL^− 1^ Zn and decreased with further increase in Zn concentration. The decarboxylated cannabinoids (THC, CBD, CBC, THCV) were reduced with Zn addition except THC and CBD in the secondary inflorescence that responded with a maximum curve with the highest concentration under 0.35 mgL^− 1^ Zn (Fig. 3). For all the cannabinoid tested, the total amount of an individual cannabinoid produced per plant increased with Zn up to 0.35 mgL^− 1^ Zn (Fig. 4).

### Gas exchange, pigments and physiology

**Fig. 5:**
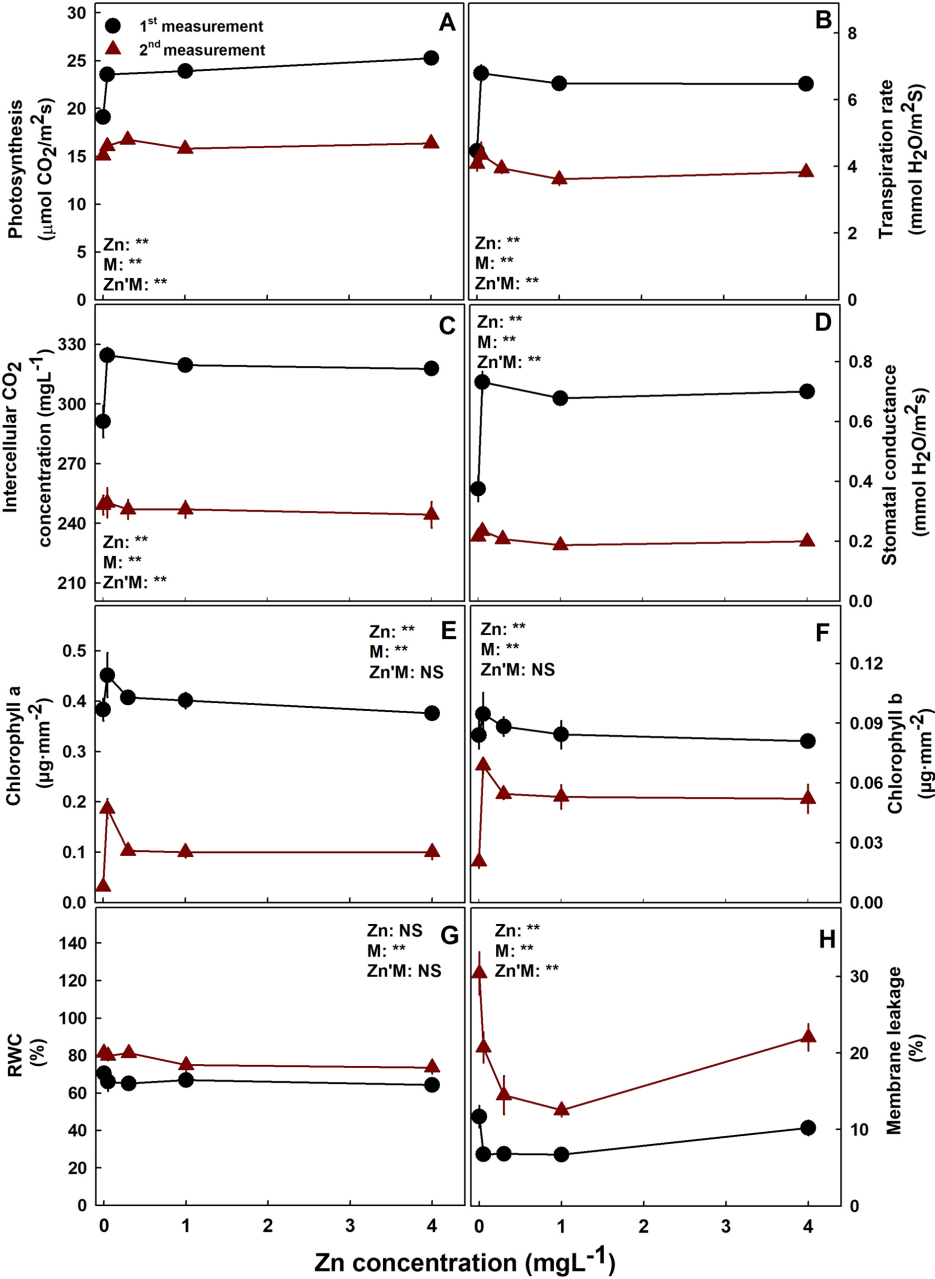
Effect of Zn supply on gas-exchange and physiological characteristics of cannabis leaves. Net photosynthesis rate (**A**), transpiration rate (**B**), intercellular CO_2_ (**C**), stomatal conductance (**D**), chlorophyll a (**E**), chlorophyll b (**F**), Relative water content (RWC) (**G**), and membrane leakage (**H**). Results of measurements at two plant developmental stages, at the middle and end of the flowering phase, conducted 36 days (1st measurement) and 14 days (2nd measurement) before harvest. Presented data are averages ± SE (*n* = 6). Where not visible, the SE are smaller than the symbol size. Results of two-way ANOVA indicated as NS (not significant) *P >* 0.05, ** *P* *<* 0.05, *F*-test. In the ANOVA results Zn’M represents the interaction between Zn and measurement

Photosynthesis, transpiration rate, intercellular CO_2_ concentration and stomatal conductance were lower under 0.05 mgL^− 1^ Zn supply at the first measurement (five weeks before harvest) then under all other Zn treatments (Fig. 5). At a later stage of plant development (at the second measurement- two weeks before harvest), the tissue was much less active, gas exchange parameters were lower and were not significantly affected by the Zn treatments and chlorophyll concentrations were lower as well compared to earlier stages of development. Unlike the gas exchange response to the Zn treatments, chlorophyll a and b concentrations were lowest under the lowest Zn supply then in all other treatments only at the second measurement. Both gas exchange parameters and chlorophyll contents reduced significantly with plant development from the 3rd to the 6th Week of the flowering phase. Membrane leakage responded with a minimum curve with the lowest values under 0.1–1.1 mgL^− 1^ Zn, and RWC was not affected by the Zn treatments at the two developmental stages tested. At the second measurement, two weeks before harvest, the leaf RWC was higher than earlier in development.

### Minerals

**Fig. 6:**
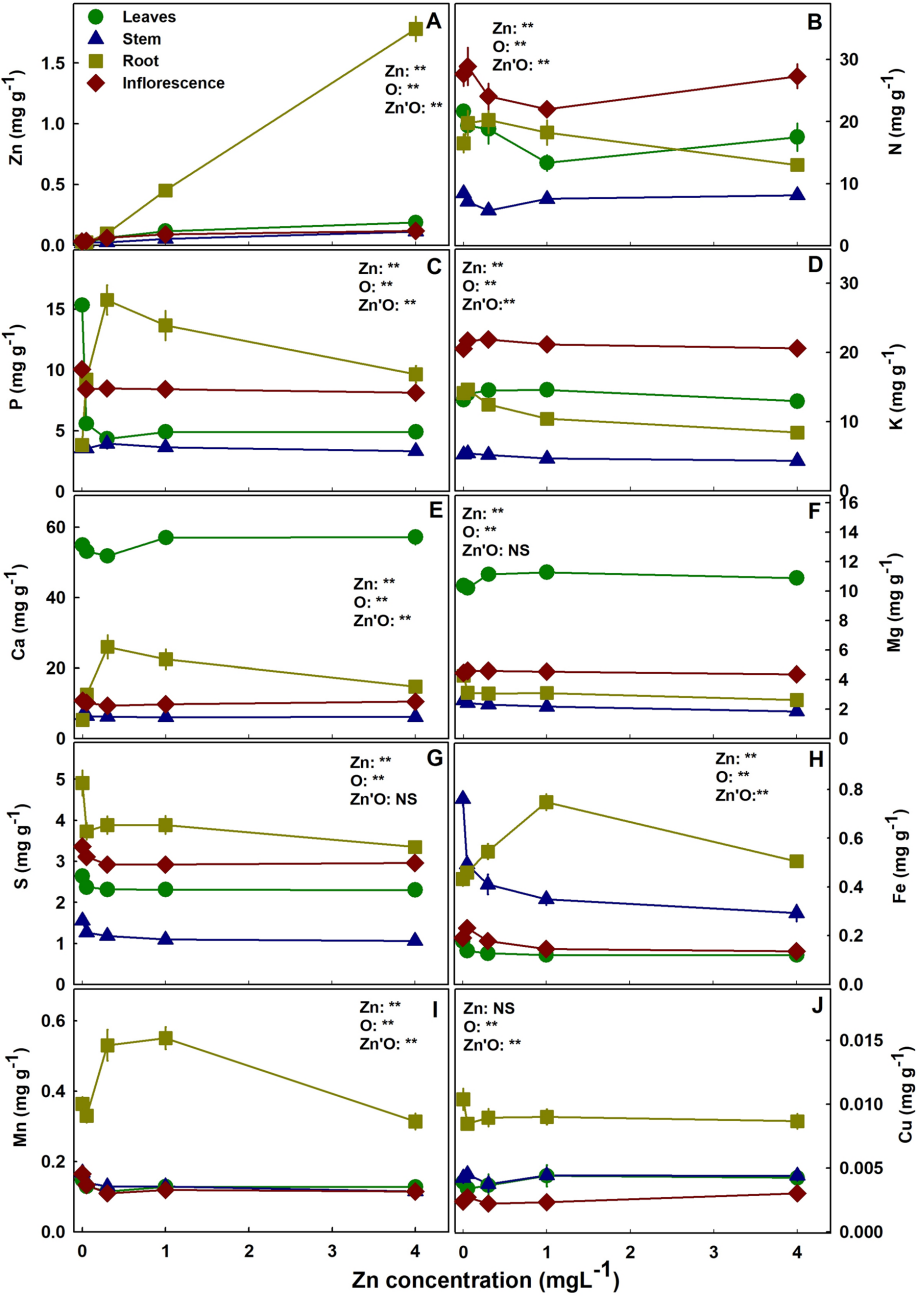
Effect of Zn supply on nutrient concentration in leaves, stem, roots and inflorescences of medical cannabis. Zn (**A**), N (**B**), P (**C**), K (**D**), Ca (**E**) Mg (**F**), S (**G**), Fe (**H**), Mn (**I**). Cu (**J**). Presented data are averages ± SE (*n* = 5), concentrations are mg/gDW^− 1^. Results of two-way ANOVA indicated as NS (not significant) *P >* 0.05, ** *P* *<* 0.05, *F*-test. In the ANOVA results Zn is zinc, O is organ, and Zn’O represents the interaction between Zn and O. Where not seen the SE are smaller than the symbol size

The concentration of Zn in all plant organs increased significantly with the increase in Zn supply (Fig. 6A). Most Zn was stored in the root, where the increase in Zn concentration was the most profound. Nitrogen in leaves, stem, and inflorescence responded in a minimum curve with the lowest concentration at 0.35–1.35 mgL^− 1^ Zn (Fig. 6B). Root’s N concentration had the opposite trend -a maximum curve response with a maximum at 0.35 mgL^− 1^ Zn. Phosphorus in leaves was highest at the 0.05 mgL^− 1^ Zn treatments (Fig. 6C), and in the roots similar to N it demonstrated a maximum curve with the highest concentration under 0.35 mgL^− 1^ Zn. Potassium concentration in the root declined with Zn addition. Magnesium was slightly lower under 0.05–0.1 mgL^− 1^ Zn in the leaves and higher in the root at 0.05 mgL^− 1^ Zn comparted to the other Zn treatments (Fig. 6F). Calcium concentration responded with a minimum curve in the leaves and a maximum curve in the root at 0.35 mgL^− 1^ Zn (Fig. 6E). Sulfur concentration in all plant organs was highest under 0.05 mgL^− 1^ Zn (Fig. 6F). Iron in shoot organs decreased with Zn supplement (Fig. 6H). Manganese in the shoot accumulated to the highest concentrations under 0.05 mgL^− 1^ Zn (Fig. 6I). In the root, Mn and Fe increased with Zn up to 0.35 mgL^− 1^ Zn and 1 mgL^− 1^ Zn, respectively and decreased at 4 mgL^− 1^ Zn.

**Fig. 7:**
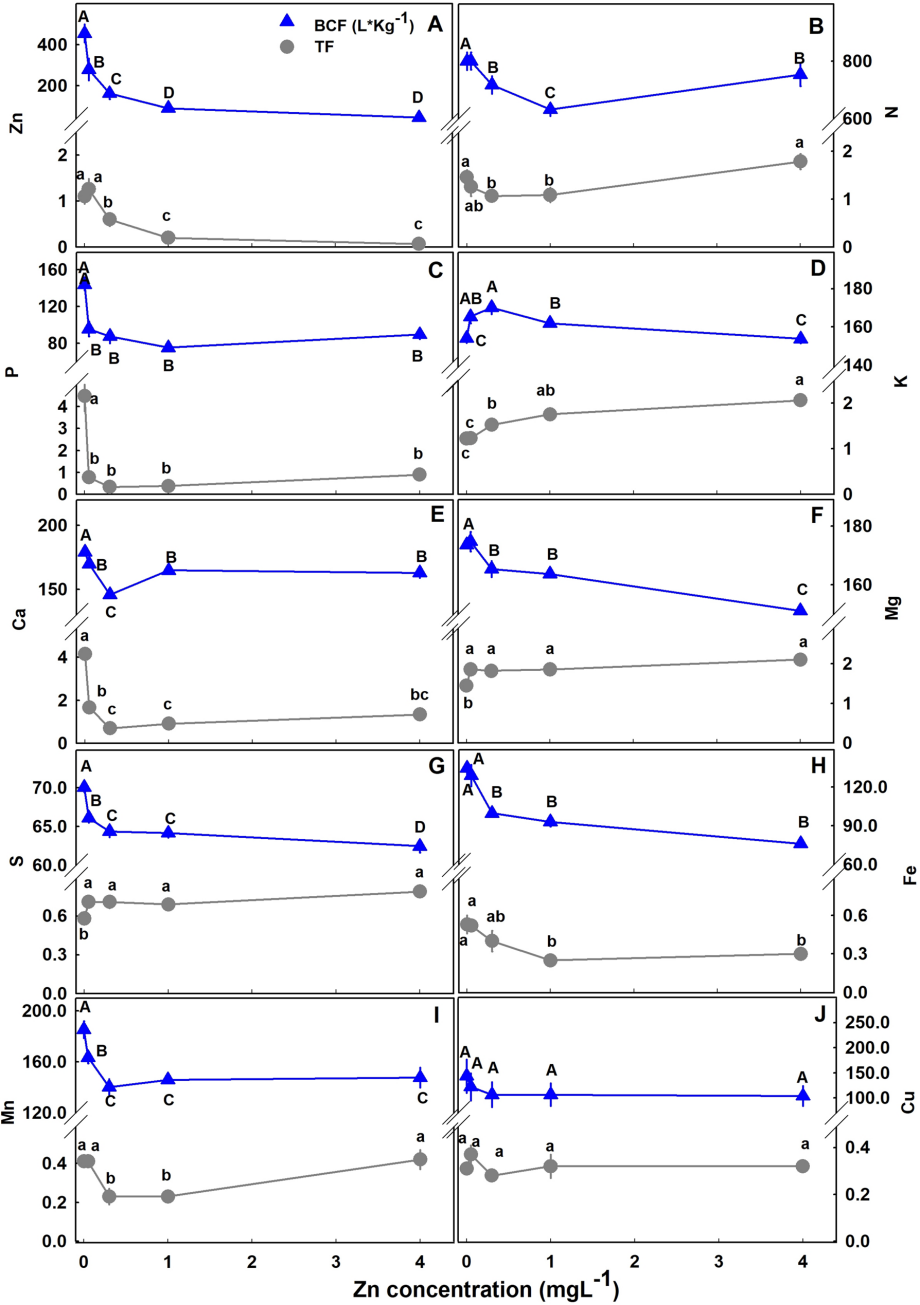
Bioaccumulation coefficient factor (BCF) (L Kg-^1^), and Translocation factor (TF) of minerals in medicinal cannabis. Within each factor curve, different letters above the means represent significant differences by Tukey HSD test (α = 0.05)

**Fig. 8:**
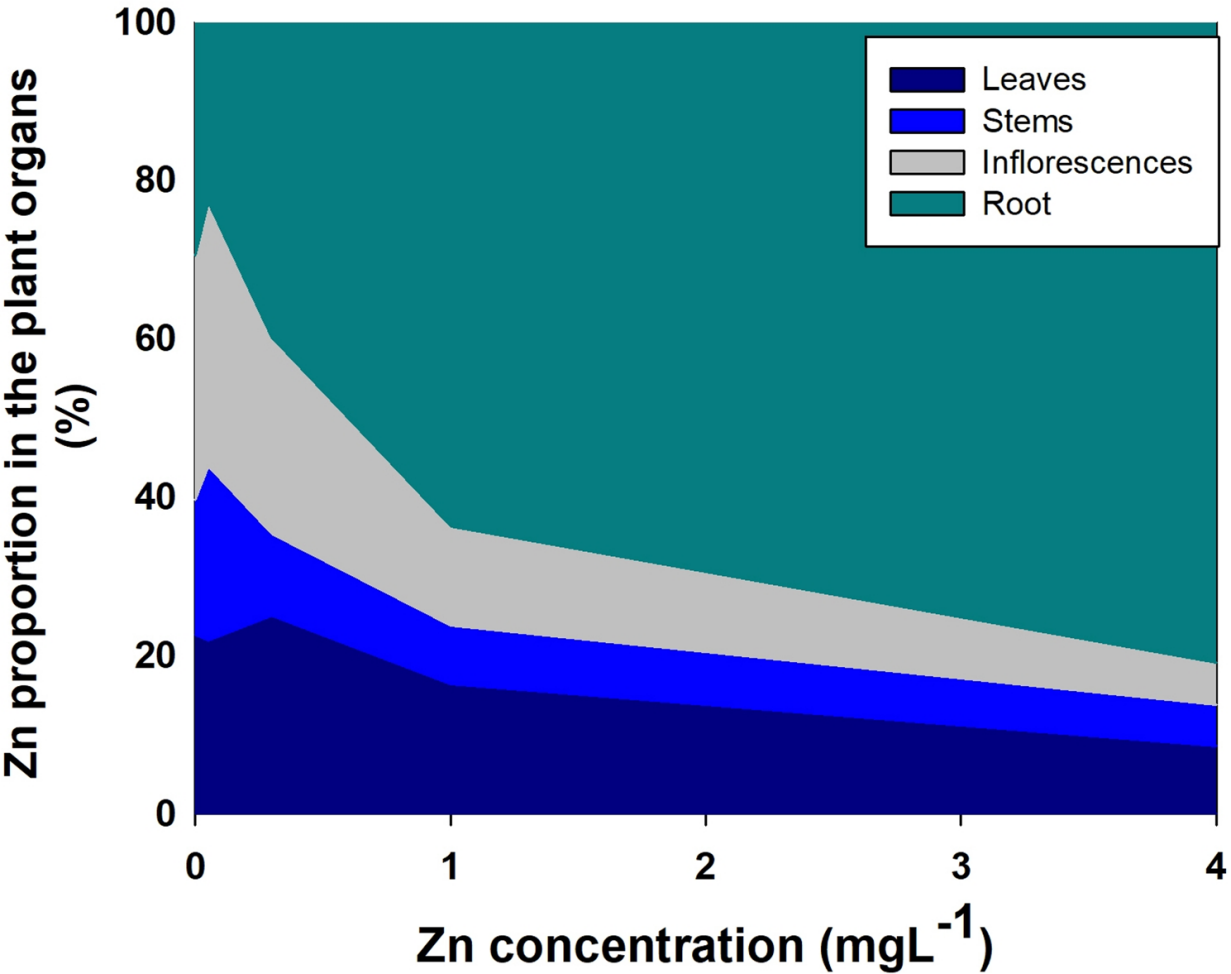
Effect of Zn supply on the distribution of Zn in medicinal cannabis plants to leaves, stems, roots and inflorescences. The total Zn content in each organ is presented as the percent content of the total Zn in the plant. Presented data are averages (*n* = 5)

The translocation of minerals from the root to the shoot as was estimated by the translocation factor (TF) was highly affected by Zn availability (Fig. 7); and so was the bioaccumulation of minerals in the plant as was estimated by the bioaccumulation coefficient factors (BCF). The BCF declined with the increase in Zn supply for Zn, P, Mg, Mn, S, Fe, with a minimum curve for Ca and N, and a maximum curve for K. The TF increased for K, S and Mg, decreased for Zn, P and Fe, had a minimum curve response for N, Ca and Mn. The TF and BCF for Cu were not affected statistically by Zn supplement. TF lower than 1 represents low translocation from root-t-shoot and a localization of the mineral in the roots. TF < 1 was found for S, Fe, Cu and Mn. A quantitative analysis of the distribution of Zn to the plant organs showed that most of the Zn in the plant accumulated in the root, and the proportion of Zn in the root increased with the increase in Zn supply (Fig. 8).

## Discussion

### Morphology, biomass and visual symptoms

Zn deficiency and toxicity symptoms in plants are known to include growth inhibition, small leaves, chlorosis, lower chlorophyll content and reduced photosynthesis rate (Robson 1993; Kaya et al. 2000; Rout and Das 2009). We report here that in medicinal cannabis as well, growth restriction was induced in plants grown under Zn deficiency (Fig. 2A). The inhibition of growth was apparent from day 32 of the treatments when elongation rate begun to decline (Fig. 2B). The restricted morphological development under 0.05 mgL^− 1^ Zn and of biomass production under 0.05–0.1 mgL^− 1^ Zn suggest that 0.35 mgL^− 1^ Zn is the optimal concentration for yield production. Stunted growth under Zn deficiency was reported for numerous plants including gladiolus (Akter et al. 2017), wheat and cotton (Cakmak and Marschner 1988).

In-spite of the growth restriction under Zn deficiency, the number of nods on the main stem was not affected (Fig. 2C), which is an indication of shorter internodes. Auxin deficiency-like response such as internodes shortening (“rosetting”) and smaller leaf area due to Zn deficiency is believed to be related to a participation of Zn in regulation of indoleacetic acid (IAA) metabolism (Broadley et al. 2007, 2012). While suppression of shoot and root growth are common Zn-deficiency sypmtoms (Broadley et al. 2007, 2012), we found biomass reduction under Zn limitation in cannabis only in the inflorescenses and not for the other plant organs (Fig. 2E). Reduction in flower yield production under Zn limitation is considered to be related to an increase in abscisic acid which causes flower abscission (Brown et al. 1993). Zn supplementation was indeed reported to increase flowering in numerious crops (Akter et al. 2017).

Unlike the effects on shoot growth, we found no effect of the Zn treatments on root growth and color (Fig. 1C). In accord with the lowest sensitivity of the cannabis roots compared to the shoots to Zn-deficiency, in other plants as well shoot growth under insufficient Zn tended to be depressed more than the root (Zhang et al. 1991). Yellow-brown color of root is a symptom of Zn toxicity in some plants (Kaya et al. 2000), however in others, the color doesn’t change (Cakmak and Marschner 1988). Chlorosis (“mottle leaf”) of young leaves is a common symptom of Zn scarcity, and death of the leaf tip is a known symptom of Zn toxicity (Rout and Das 2009) as we have found for cannabis as well (Fig. 1). Cockson et al. (2019) found that Zn deficiency in hemp first appeared as marginal chlorosis in new and expending leaves.

### Cannabinoid production

Quantity of secondary metabolites in a plant is the integrated balance between production, and loss by catabolism. It is affected by genetic and environmental conditions. Recent work with medicinal cannabis revealed a substantial influence of N (Saloner and Bernstein 2021b), P (Shiponi and Bernstein 2021b), Mg (Morad and Bernstein, 2025) and K (Saloner and Bernstein 2022) availability, NH_4_/NO_3_ ratio (Saloner and Bernstein 2021a) as well as an integrated effect of N, P, and K supply (Bernstein et al. 2019b; Bevan et al. 2021), and restricted fertigation pre-harvest (Saloner et al., 2024) on the cannabinoids profile. Zn nutrition was found to have a substantial influence on cannabinoids concentration, and the dose-response trends were mostly similar for the two apical inflorescences tested from the top and bottom of the plants (Fig. 3). The concentrations in the bottom [secondary] inflorescences were lower than at the top ones in all treatments, in agreement with previous reports (Bernstein et al. 2019a; Danziger and Bernstein 2022; Saloner and Bernstein 2021b). Interestingly, the acidic (carboxylated) cannabinoids and the non-acidic (decarboxylated) cannabinoids responded differently to the Zn treatments. The highest concentration of acidic cannabinoids was achieved under 0.1–0.35 mgL^− 1^ Zn, while for the non-acidic cannabinoids the highest concentration was reached under 0.05–0.1 mgL^− 1^ Zn. Zn scarcity (0.05 mgL^− 1^ Zn) and excess (1–4 mgL^− 1^ Zn) reduced the concentrations of all acidic cannabinoids. A differential response of the acidic versus the decarboxylated cannabinoids to mineral nutrition was demonstrated before for N (Saloner and Bernstein 2021b) and P nutrition (Shiponi and Bernstein 2021b), and results from deficiency/toxicity stress effects on decarboxylation.

Zn supplement was demonstrated to increase essential oil production and effect its composition in other medicinal plants and herbs as well including cumin (El-Sawi and Mohamed 2002), plants from the Lamiaceae family (Hegazy et al. 2016) and Pelargonium (Misra et al. 2005). In medicinal cannabis, a positive effect of Zn supply on cannabinoid production was apparent up to 0.35 mgL-1 Zn supply, and further Zn addition resulted in a decline in concentrations of all the cannabinoids tested (Fig. 3). This is in accord with a reported reduction of essential oil production under excess Zn in *Pelargonium graveolens* (Misra et al. 2005).

Regardless of the maximum dose-response curves of cannabinoid concentrations to Zn supply, the total production of cannabinoids per plant increased with Zn supply up to 0.35 mgL^− 1^ (Fig. 4), reflecting the increase in the inflorescence’s biomass. A similar trend of increased cannabinoids production per plant despite the decrease in concentration was obtained also for P nutrition (Shiponi and Bernstein 2021b). P and Zn are well known to have antagonistic effects for plant uptake and translocation of one another (Soltangheisi et al. 2013). We reported a similar response also for medical cannabis (Shiponi and Bernstein 2021a,b), (Fig. 6). It might be argued that the identified reduction in cannabinoid concentrations is due to Zn influence on the P status of the plant. However, P effect on cannabinoid concentrations negatively correlated with yield production and was suggested to result from a dilution effect (Shiponi and Bernstein 2021b). In the present study no such correlation was found, and Zn effect on cannabinoid concentrations was via different mechanisms. The reduced concentration under Zn deficiency may have resulted from Zn-induced P toxicity. Yet, effect of high P supply on the cannabinoid concentrations was limited, and stimulation of cannabinoid production by P supply occurred mostly under low P (Shiponi and Bernstein 2021b). However, the leaves’ P concentration in the P study did not exceed the P concentration in Zn deficient leaves, and the effect of the considerably higher (~ X3) P concentration found in leaves of the Zn-deficient plants on cannabinoids production is unknown.

Environmental stresses are well known to affect secondary metabolite production in plants, and has been used for elicitation of secondary metabolite production in medicinal plants (Gorelick and Bernstein 2014). The negative effect of Zn on cannabinoid production may be induced by stress, since under low and high Zn supply membrane leakage (a sign of tissue stress) was increased (Fig. 5H) in parallel to the reduction of cannabinoids accumulation. The stress-related molecules, salicylic acid and GABA (Gamma aminobutyric acid) were indeed found to elicit changes in the cannabinoids biosynthesis pathways by affecting expression of key enzymes and ultimately the cannabinoids content (Jalali et al. 2019). Further research is needed to elucidate the interrelations between stress conditions and the effect of mineral nutrition in general and Zn nutrition in particular on cannabinoid metabolism. Zn is associated with metabolic pathways in the cell as a cofactor of a large number of enzymes. It can bind to proteins to form zinc metalloenzymes, and thus involve in various biological processes including transcription, translation, photosynthesis, and metabolism of reactive oxygen species (ROS) in plants.

Zn play a role in activity of numerous enzymatic classes (including lyases, oxidoreductases, hydrolases, transferases, ligases, and isomerases) and has many functions in regulatory proteins (Bouain et al. 2014). It is a catalytic and structural protein cofactor in hundreds of enzymes and has key functions in the domains that interact with other molecules; and the Zn-finger proteins mediate DNA binding of transcription factors and protein–protein interactions. Zinc finger-homeodomain (ZF-HD) transcription factors also act as molecular switches to fine-tune metabolic pathways, thus regulating secondary metabolism, controlling production of compounds like flavonoids and terpenes, by activating/repressing related biosynthetic genes (Shen et al. 2025). For example, in relevant to medicinal and aromatic plants, the WRKY transcription factors that contain a zinc-finger region, regulate the biosynthesis of several secondary metabolites including phenols, lignin, flavonoids and tannins, and WRKY3 and WRKY6 are involved in the biosynthesis of terpenes (Shen et al. 2025). It is therefore not surprising that Zn was found to affect the accumulation of secondary metabolites in plants including terpenes and flavonoids (Fan et al. 2021; Zhang et al., 2024), two of the main secondary metabolite groups produced also by cannabis. No information is currently available about the effect of Zn nutrition on metabolism of cannabinoids and other secondary metabolites in cannabis. Future research should explore the functions and regulatory mechanisms of Zn related transcription factors and proteins in in cannabis using multi-omics analysis and molecular biology techniques.

Zn deficiency disrupts enzymes activity that can cause quality and yield reduction (Alloway 2008). The reduced cannabinoid concentration under Zn stress may be caused by a direct or indirect interference with enzyme activities that disturb cannabinoids biosynthesis or catabolism. Although the mechanism by which Zn effects secondary metabolites accumulation in medicinal cannabis is not yet known, our results revealed that Zn nutrition can be used to elicit changes in the cannabinoid profile in the plant and thus for regulation of the medicinal quality. These results point yet again at the potential of mineral nutrition for fine-tuning the medical quality of the cannabis product and emphasize the importance of increasing our understanding of cannabis mineral nutrition, for generating the knowledge needed for development of precision fertilization.

### Physiological responses

Zinc is essential for the maintenance of biomembranes’ integrity. It has significant roles in cell defense against free radicals by the regulation of both generation and detoxification of the free oxygen radicals (Broadley et al. 2012). It functions as a co-factor in activating many important antioxidant enzymes in plants (Marreiro et al. 2017) thus reducing oxidative damages. It is therefore not surprising that ROS accumulates in Zn deficient plants, leading to oxidative damage and an increase in plasma membrane permeability (Sadeghzadeh 2013), as was observed also for cannabis in our study. Zinc controls the ROS status of the cell by playing a key role as a component of CuZnSOD, interfering with the oxidation of NADPH oxidase and indirect involvement in H_2_O_2_ scavenging enzymes activity (Cakmak 2000). The binding of phospholipids to the membrane’s sulphydryl groups further contribute to the membrane’s integrity (Sadeghzadeh 2013). However, excessive concentrations of zinc can overload the antioxidant defense system, resulting in decreased plant antioxidant activity which increases membrane permeability, lipid peroxidation and reduces sulphydryl content (Madhava Rao and Sresty, 2000; Tripathi and Gaur 2004). Accordingly, in the cannabis plants as well, the high level of membrane leakage obtained under high and low Zn (Fig. 5H) indicates oxidative damage and lipid peroxidation (Bernstein et al., 2010) the high level of membrane leakage obtained under high and low Zn (Fig. 5H) indicates oxidative damage and lipid peroxidation (Bernstein et al., 2010). In the mature plants Chlorophyll a and b contents were lower under Zn deficiency (Fig. 5E, F), as was reported for other plants (Hu and Sparks 1991). The low chlorophyll content under low Zn can result from chloroplast degradation caused by reduced ROS-scavenging enzyme activity in the chloroplast (Cakmak 2000). Interestingly, although oxidative stress was suggested in the mature plants under deficiency and toxicity of Zn (by the increase in membrane leakage and the decrease in chlorophyll concentration), photosynthesis was not affected by the treatments at this stage of development (Fig. 5). The lack of response of the gas exchange parameters in the mature plants may be related to the advanced senescence at this stage of development that was accompanied also by a reduction in photosynthesis in all treatments. This notion is reinforced by the lower values of photosynthesis, transpiration rate, stomatal conductance, intercellular CO_2_ and chlorophyll content compared to the earlier stage of development.

Zn deficiency is well known to reduce photosynthesis by few mechanisms: (1) inhibition of Carbonic Anhydrase activity, (2) effect on other photosynthesis key enzymes such as ribulose 1,5-bisphosphate carboxylase (RuBPC), (3) reduced chlorophyll content, (4) modifications of the chloroplast structure (Randall and Bouma, 1973; Brown et al. 1993; Sadeghzadeh 2013). Since no significant decrease in chlorophyll concentration was found at the first measurement, the lower photosynthesis under Zn insufficiency at this stage most likely reflects an effect on photosynthetic enzymes, and only later in development, the reduction in chlorophyll concentration contributed to this inhibition.

It was demonstrated for numerous plant species that excess Zn reduces photosynthesis and chlorophyll content (Tsonev and Lindon 2012), likely duo to a replacement of Mn by Zn in the water splitting complex, and substitution of Mg by Zn in the chlorophyll molecule and reduction in chlorophyll synthesis and stimulation of chloroplast degradation (Broadley et al. 2007). In the current experiment, photosynthesis and chlorophyll content were not affected by high Zn supply. This may be due to Zn retention in the root rather than in the aerial parts of the plant (Fig. 6). Accumulation in the underground tissues is an indirect way to protect the photosynthetic shoot from toxicity damage (Gupta et al. 2016).

### Zn accumulation and distribution in the plant

Plants acquire Zn mainly in the form of divalent ion. Zn acquisition by the roots and distribution to the plant tissues is affected by root morphology, leaf number, biomass, age, developmental stage, soil physical and chemical properties etc. (Gupta et al. 2016). The bioaccumulation coefficient (BC), also known as the biological absorbent coefficient (BAC) or the bioconcentration factor (BCF), is a common factor used to evaluate uptake of a metal by the ratio of the concentration in the plant and in the growing medium (Phetsombat et al. 2006; Doucette et al. 2018). The BCF of Zn was significantly reduced with the increase of Zn in the nutrient solution (Fig. 7A). According to Michaelis-Menten kinetics for active uptake, the flux of an ion into the plant depends on it’s concentration in the external solution. Under low external nutrient concentration, the influx of an ion increases until saturation is reached. Under low external concentrations most nutrients are taken up by high affinity selective transporters that require energy input to balance the acquisition to the plants demand. Efficient acquisition of a nutrient under low Zn, and reduced uptake under elevated Zn (Fig. 7A) demonstrate regulation of the Zn status in cannabis. Regulation of metal accumulation is vital for plants and is achieved by coordination of the uptake, accumulation and *in-planta* translocation processes.

Zinc distribution within the plant was affected by the level of Zn supplied (Fig. 8). Translocation factor (TF) is widely used to describe the translocation of a substance from the root to the shoot (Lesmeister et al. 2021). The TF values show that the translocation from root to shoot decreased with the increase of Zn in the nutrient solution (Fig. 7A). Zn compartmentation in the root is a defense mechanism against oxidative damage to the photosystem (Gupta et al. 2016). P concentration in the nutrient solution used in the current study is relatively high (60 mgL^− 1^), as is common in medicinal cannabis production (growers supply up to 200 mgL^− 1^ P) (Shiponi and Bernstein 2021a, b). Under high Zn conditions, compartmentation of Zn in the root can take place due to the formation of Zn-Phytate (Loneragan and Webb 1993). Zn localization in plants depends upon genetic, biotic and abiotic conditions such as the external Zn concentration and it’s interaction with other metals. For many plants, Zn concentration in the shoot is lower than the root (Gupta et al. 2016). In cannabis, under low Zn supply, we found that a high proportion of the Zn taken up by the plant is translocated to the shoot to satisfy the shoot demand, and the proportional accumulation in the root is lower than under higher Zn supply (Figs. 7 and 8).

Biomass must be considered when accumulation is being evaluated. For example, the increased Zn concentration in bush bean’s roots was due to a decrease in root growth (Ruano et al. 1987). In spite of the increase in shoot biomass with the increase in Zn supply (and the lack of change in root biomass) (Fig. 2), the proportion of Zn which accumulate in the root out of the total Zn biomass in the plant increased with the increase in Zn supply (Fig. 8). We therefore conclude that the effect of the Zn treatments on Zn concentration in cannabis roots is due to specific localized Zn accumulation and not a dilution effect (Fig. 2). The increase in the relative Zn content with Zn supply supports this conclusion (Fig. 8). Zinc concentration in the root is an integrated result of regulation of the expression and the activity of Zn transporters (Gupta et al. 2016). Compartmentation of Zn in the root vacuole is a mechanism of Zn homeostasis in the plant that provide resistance to Zn toxicity (Sinclair and Krämer 2012). Toxicity of heavy metals such as Zn and Cu in plants can be caused by binding of the metal to sulfhydryl groups of proteins that can induce conformation changes and inhibition of protein activity, in addition, excess of heavy metals elicits ROS production and thereby stimulate oxidative stress (Hall 2002). Zn was found to be stored in subcellular compartments and chelated to organic acids (Samardjieva et al. 2015b), and cell wall (Longnecker and Robson 1993; Clemens et al. 2002). Understanding the mechanistic effects of Zn toxicity and Zn tolerance in cannabis can benefit from further studies into mechanism of Zn compartmentation in cannabis roots; specific potential effects of Zn toxicity on protein activity by specific Zn-protein binding; and effects on localized induced changes in ROS production and oxidative stress.

In spite of the strong retention of Zn in the root, no reduction in root biomass or length was found due to zinc toxicity (Figs. 1 and 2). Accumulation of Zn, Mn, Fe and Cu in the root under adequate nutrient supply was also found in cannabis at the vegetative stage (Saloner et al. 2019; 2020; Shiponi and Bernstein 2021a). Similar results were obtained when P accumulated in the root at the vegetative phase of cannabis plants under excess P in the nutrients solution (Shiponi and Bernstein 2021a). However, at the reproductive stage, the highest concentration of P was found in the inflorescence. Samardjieva et al. (2015a) reported that in *Solanum nigrum* Zn accumulation in the root was similar pre- and post flowering. In cannabis, under Zn deficiency (0.05, 0.1mgL^− 1^) a large proportion of the Zn taken up by the plant was translocated to the shoot tissues. However, the percentage of Zn in the inflorescences decreased from 30% under Zn scarcity, to 24% under optimum concentration (0.35 mgL^− 1^) to 5% under excess Zn (4mgL^− 1^) (Fig. 8). In spite of the decrease in the proportion of Zn translocated to the inflorescence, the inflorescences concentration increased with the increase in Zn supply, likely since Zn is known to be required for seed germination (Rengel and Graham 1995). It is important to note that there is a considerable genetic variability in Zn accumulation in reproductive tissues, and more information is therefore required about genotypic variability in Zn uptake and accumulation patterns in cannabis (Longnecker and Robson 1993).

Zn concentration in plants grown under adequate Zn, ranges 15–100 mg kg^−1^dw (Longnecker and Robson 1993). The threshold deficiency concentration for Zn in leaves is considered to be < 15–20 mg kg^−1^dw (Broadley et al. 2012; Sinclair and Krämer 2012). Under the insufficient Zn treatments, Zn concentration in the cannabis leaves was 14.7 and 23.5 mgkg^−1^dw for the 0.05 and 0.1 mg ZnL^− 1^ treatments, respectively (Fig. 6); and yield reduction was found for both treatments. However, for the 0.1 mgL^− 1^ treatment, no visual or physiological symptoms were identified beside biomass production (Figs. 1 and 2). Thus, both leaves’ Zn concentration are within the deficiency range, and the leaf critical deficiency threshold concentration is slightly higher than the 20 mg Zn kg^−1^dw documented in the literature for other species. The critical toxicity concentrations of Zn accepted for plants are < 100 to < 300 mgZn kg^−1^dw (Ruano et al. 1988; Broadley et al. 2012). Although Zn concentration in leaves at the 4mgL^− 1^ treatment was within the toxicity range (187.9 mgZnkg^−1^dw) no severe toxicity symptoms were found. Crops are typically very tolerant to high Zn concentrations but sensitivity to Zn toxicity vary between plants.

### Zn bioaccumulation

With the increase usage of cannabis for medicinal purposes, there is a growing concern about the safety of the plant product. Of major concern is the potential bioaccumulation of heavy metals(McPartland and McKernan 2017; Sarma et al. 2020) Including Zn. Zinc toxicity by cannabis consumption can occur following smoking, inhalation, or oral intake. Excessive oral uptake can lead to neurological symptoms and anemia, as well as impaired copper absorption (Whahab et al., 2020). Inhalation of high Zn containing smoke can induce symptoms of upper airway obstruction, consolidation and pulmonary edema (Rudy, 1994), and a prolonged exposure to zinc-containing fumes may lead to the development of metal fume fever, (Brenner and Keyes 2024), and affect lungs morphology, including eosinophilia, goblet cell hyperplasia and pulmonary fibrosis, indicating an inflammatory process (Cho et al. 2011).

Fiber-type (Hemp) cannabis is recognized as a hyperaccumulator plant and was suggested in the past for soil bioremediation (Ahmad et al. 2016; Zielonka et al. 2020). In the current study, the Zn concentrations studied did not exceed the concentrations found in contaminated soils (Kabata-Pendias and Pendias 2001). However, the high Zn treatment used in the experiment is over 10-fold the concentration used by the growing cannabis industry (~ 0.3mgL^− 1^ Zn), therefore, it is suitable for comparison and for understanding the plant bioaccumulation behavior under realistic crop exposure concentrations.

More than 450 plants are defined as metal-hyperaccumulators but only 10–20 species are reported as Zn accumulators (Broadley et al. 2007; Gupta et al. 2016). Zinc mechanisms for tolerance includes reduced uptake, compartmentation in subcellular compartments, altered translocation, accumulation in older leaves, chelation, binding to cell wall and involvements of repair and protection proteins (Hall 2002).

Hyperaccumulators of Zn are plants that contain over 1% of Zn in their dry weight (Baker and Brooks 1989). In cannabis, under 4mgL^− 1^ Zn supply our calculated average Zn concentration in the whole plant was ~ 0.007%. However, testing the plant potential as a hyperaccumulator requires further study. Unlike Zn localization in cannabis roots, in most hyperaccumulating plants, Zn is stored mostly in leaf’s vacuoles and the translocation to the shoot is enhanced (Broadley et al. 2007; Sinclair and Krämer 2012; Gupta et al. 2016). Malik et al. 2010 reported for Zn in wild cannabis a TF > 1, that suggest high translocation of Zn to the shoot and potentially the smokable tissues. In the current experiment TF > 1 under Zn restriction was also obtained for Zn, however with increasing Zn availability, TF declined and was lower than 1 which represent root localization. Zn accumulation in the root cortex and in leaves is common in tolerant plants under toxic Zn supply (Longnecker and Robson 1993). In many plants, the root’s vacuoles function as the main storage site for excess Zn in the plant (Sinclair and Krämer 2012). Although hemp plants were found to increase heavy metal transfer from root to shoot under excess metal stress (Ahmad et al. 2016), Zn tend to accumulate in hemp’s root (Shi and Cai 2010; Rheay et al. 2021). When tested for it’s phytoremediation potential, Hemp was shown to accumulate Zn in the plant tissues in the order: inflorescences> leaves> root> stem (Pietrini et al. 2019). Accumulation of Zn in the roots was found in 4 hemp cultivars grown hydroponically under toxic concentration of Zn (Petrova et al. 2005). Since, the bioaccumulation potential of plants varies between and within species and cultivars (Petrova et al. 2005), and medical cannabis and hemp were bred for different purposes, the accumulation characteristic may be significantly different. Therefore, assessment of medicinal cannabis’ metals bioaccumulation and its concentration in the inflorescences needed to be tested separately from hemp.

Based on the organs of heavy metal accumulation (in shoot vs. roots), plants are classified as metal accumulators or excluders. Accumulators’ strategy for survival under toxic heavy metals concentration is metal storage in the leaf vacuoles, and excluders’ strategy is localization in the root (Baker 1981). Our results show that the cannabis tactic for tolerance is mainly exclusion, and therefore accumulation in the shoot was less affected. Under such exclusion scenario, the effect on the pharmacological product is small.

### Effect of the Zn treatments on mineral uptake and distribution

Zn affects uptake and transport of minerals in the plant. Interaction with N, K, Ca, Mg, S, Fe, B, Mn and Cu were reported for many plant species (Alloway 2008; Rout and Das 2009; Prasad et al. 2016). Welch and Norvell (1993) suggested that the effect of Zn on mineral uptake and translocation involves protection of the sulfhydryl groups of the plasma membrane proteins from oxidation.

The interaction between P and Z uptake in plants is well documented. In cannabis, Zn deficiency had a substantial effect on P concentration in leaves, P uptake into the plant and root-to-shoot translocation (Figs. 6B and 7B). The crosstalk between P-Zn was termed “P induced Zn deficiency” and numerus studies demonstrated the negative interaction between these two essential elements on various crops Loneragan and Webb 1993; Zhu et al. 2001; Soltangheisi et al. 2013). Accumulation of P in Zn deficient cannabis’ leaves was significant and was accompanied by a decrease in P concentration in the root (Fig. 6B). Accumulation of P in the shoot was the result of enhanced uptake (BCF) and translocation from root to shoot (TF) (Figs. 7 and 8). In potato as well, excess P was found in Zn deficient leaf tissue as a result of enhanced uptake and translocation to the shoot (Christensen and Jackson 1981), and increased P uptake in Zn undersupplied plants was found also in cotton (Cakmak and Marschner 1986).

Cycling of P from shoot to root is impaired under Zn scarcity (Gupta et al. 2016). In wheat, P export from older leaves was depressed (Longnecker and Robson 1993). Zn deficiency enhanced P uptake and translocation up to toxic concentration in corn leaves (Soltangheisi et al. 2013). Phosphorus concentration in leaves of Zn deficient plants under high P supply increased up to toxic concentration (Loneragan, 1982), and in many plants low Zn induced uncontrolled uptake of P that increase leaf symptoms. The P concentration under 0.05 mgL^− 1^ Zn in medicinal cannabis leaves reached 15.6 mg g^− 1^, which is within the reported range of toxicity in leaves (11–45 mg g^− 1^) (Loneragan et al. 1982). The high P concentration may be the reason for the development of symptoms in the leaves of the 0.05mgL^− 1^ Zn treatment and not the 0.1mgL^− 1^ Zn, in spite the small difference in leaves’ Zn concentration between the two. Hence, the plant’s visual symptoms may represent P toxicity in leaves rather than direct Zn deficiency symptoms. In former studies, we tested the effect of high P concentration on medicinal cannabis and no visual toxicity symptoms were obtained (Shiponi and Bernstein 2021a, b). However, most P accumulated in the inflorescences and in the root at the reproductive and the vegetative growth phases, respectively and the highest concentration achieved in leaves in those studies was small (5.8 mg g^− 1^) compared to the high P concentration obtained under Zn scarcity (15.6 mg g^− 1^).

Evidence show that P-induced Zn deficiency is a feedback control mechanism and that several genes induced by Zn deficiency are repressed by P deficiency (Khan et al. 2014). Regulation of Pi homeostasis in the plant involves numerous genes, and Zn deficiency repress and induce genes related to Pi uptake and distribution (Khan et al. 2014). For example, Zn status in barley was found to be linked to the expression of the high affinity P transporters HVPT1 and HVPT2 (Huang et al. 2000), and the expression of genes of the Pht1 gene family that codes for Pi transporters was affected by Zn starvation)Jain et al. 2013(.

Competitive inhibition between Cu and Zn for plant uptake was documented for many plants (Rout and Das 2009). The lack of effect of the Zn treatments on Cu can be explain by the reduced competition between the metals due to their supply as chelated and the cultivation in soilless culture.

In medical cannabis, we demonstrated an influence of Zn on the uptake and translocation of numerous mineral nutrients in the plant (Fig. 7). Increased uptake under Zn shortage was found for P, S, Ca, Mg, Fe and Mn, and the opposite interaction was not found. The consistent response of increased uptake for most of the mineral nutrients tested, together with the strong influence on the translocation, demonstrate the loss of uptake regulation as a result of insufficient Zn.

In conclusion, the cannabis plant responded to Zn deficiency with stunted growth, chlorosis, and reduced concentration of the major cannabinoids. Zn accumulated preferentially in the plant roots and high Zn supply reduces the relative accumulation in the inflorescences and therefore increasing Zn supply does not impose further risk to the consumers. Zn deficiency increased the uptake of P, S, Ca, Fe, Mn and Zn into the plants. Phosphorus accumulated in the shoot to high concentrations under Zn deficiency due to increased uptake and translocation. Excess Zn did not result in sever toxicity symptoms, and plant production was not affected. However, cannabinoid concentrations decreased under high Zn supply. This study revealed that the optimal Zn concentration for maximum yield and cannabinoids production is 0.35 mgL^− 1^. Therefore, the recommended Zn concentration in the fertigation solution to be used in the production filed, for facilitating high specialized-metabolite production, and optimal yield and plant performance is 0.35 mgL^− 1^.

## Data Availability

All data is available in the manuscript.
